# A Rare Cause of Chronic Flexor Tenosynovitis of the Finger

**DOI:** 10.7759/cureus.40175

**Published:** 2023-06-09

**Authors:** Sei Haw Sem, Wang Chen Kang

**Affiliations:** 1 Orthopaedics, Hospital Sultanah Aminah Johor Bahru, Kuala Lumpur, MYS; 2 Faculty of Medicine, University Sultan Zainal Abidin, Kuala Terengganu, MYS; 3 Orthopaedic Surgery Department, University Malaya Medical Centre, Kuala Lumpur, MYS

**Keywords:** finger, bartonella henselae, granuloma, flexor tenosynovitis, cat scratch disease

## Abstract

Cat scratch disease (CSD) is an uncommon condition. When a patient is infected, the disease is often self-limiting. Cat scratch disease involving the musculoskeletal system had been described, but the manifestation of the disease in hand remained unexplored. We report a case of chronic flexor tenosynovitis of the left index finger caused by cat scratch disease. In this case, the antibiotic treatment did not improve the clinical outcome. However, surgical debridement of the diseased finger resulted in a tremendous improvement in terms of pain and range of motion.

## Introduction

Domestic pets are gaining popularity in Asia. Injuries due to cat bites are more common in our current practice. Most of the patients presented late following cat bites due to poor awareness of the potential serious complications. The culture from cat bites usually reveals Pasteurella multocida [1]. Occasionally, a sinister condition called cat scratch disease (CSD) can occur. CSD is caused by the inoculation of Bartonella henselae following contact with domestic animals, such as cats. The disease has a chronic nature, and patients typically develop regional lymphadenopathy one to three weeks after inoculation. Symptoms can persist for several months. While asymptomatic cases are usually self-limiting, more severe cases may require a course of antibiotics for treatment [2,3]. Although CSD involving the musculoskeletal system has been described, its manifestation in the hand remains unexplored [4]. In this report, we present a case of chronic flexor tenosynovitis in the left index finger caused by CSD. We also discuss the challenges of diagnosing and treating this rare condition.

## Case presentation

**Figure 1:**
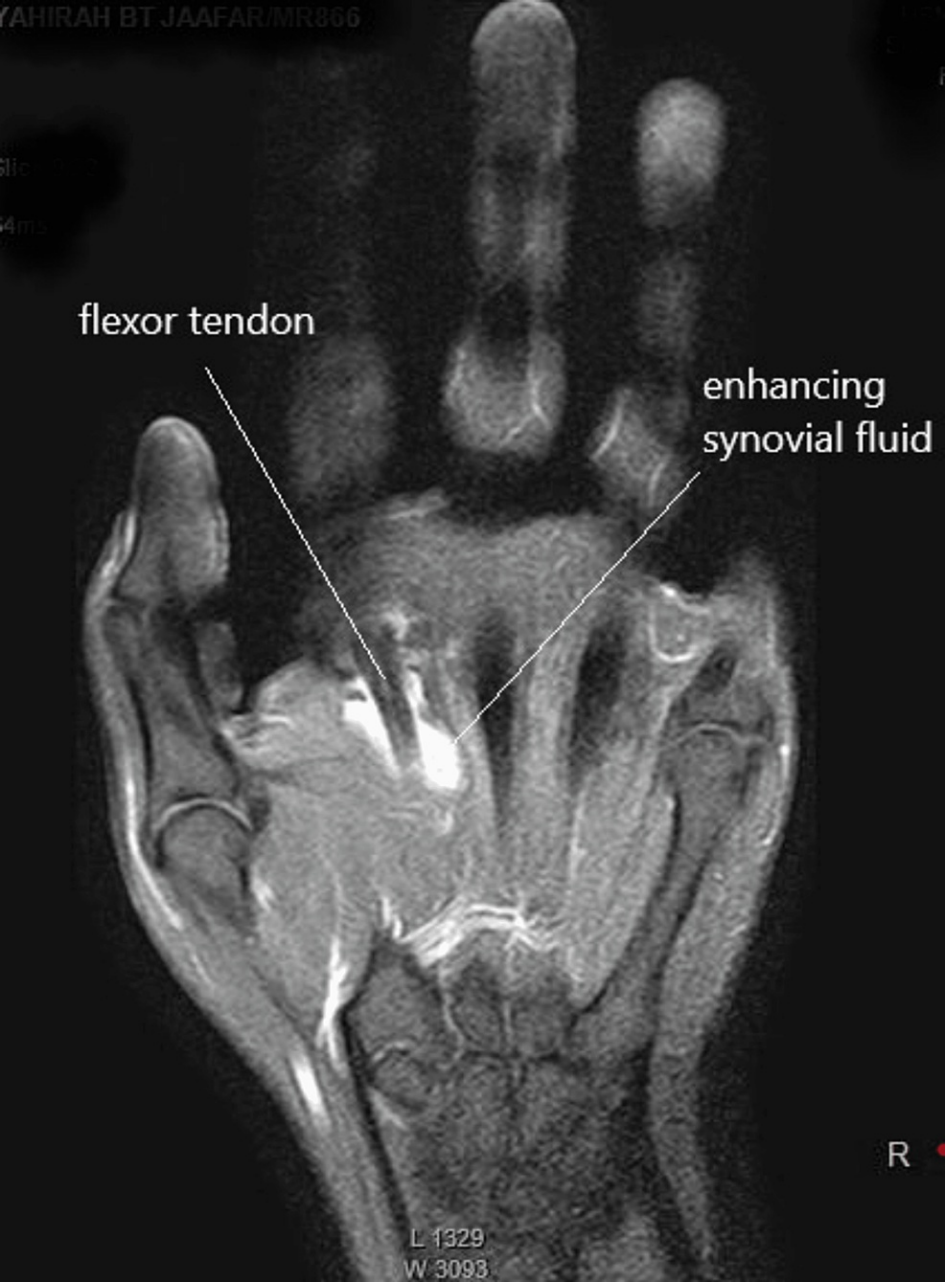
MRI coronal view of the left hand. MRI of the left index finger showed a hyper-intensified signal around the flexor tendon. MRI: magnetic resonance imaging

**Figure 2:**
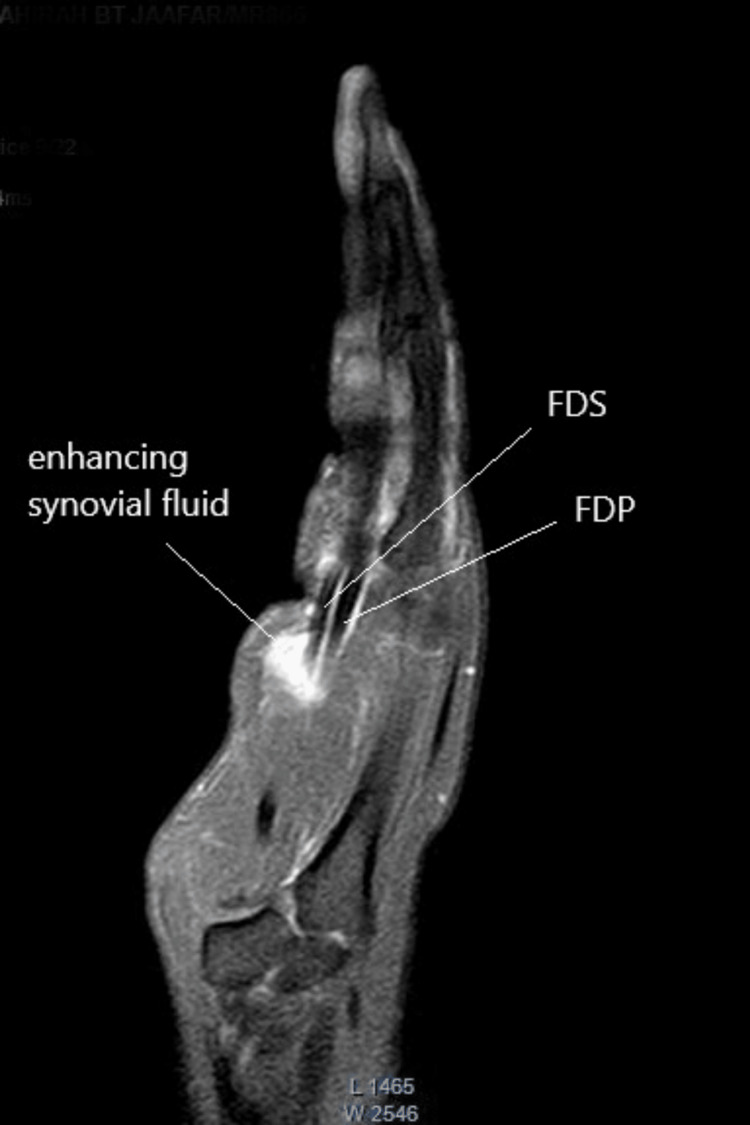
MRI sagittal view of the left hand. Note the flexor tendons remained in integrity. The enhancing signal appeared along the synovial sheath. FDS: flexor digitorum superficialis; FDP: flexor digitorum profundus; MRI: magnetic resonance imaging

A 25-year-old female with a history of diabetes mellitus complained of persistent pain and swelling in her left index finger. Over the course of one year, she completed multiple courses of antibiotics. Further investigation revealed that the symptoms began following an episode of cat scratching. She also recalled experiencing regional lymphadenopathy in the axilla on the same side one week after the cat scratch. While the ascending erythema up to the elbow resolved spontaneously, the pain in her left index finger persisted. She initially received antibiotic treatment at a district hospital for a year before being referred to a hand expert. Despite completing multiple courses of antibiotics, her symptoms did not improve. On clinical examination, diffuse swelling was observed over the volar aspect of the left index finger, along with multiple palpable nodules. Flexion and extension of the left index finger were significantly reduced. The active range of motion was 10°-20°, 30°-45°, and 20°-30° at the metacarpophalangeal, proximal interphalangeal, and distal interphalangeal joints, respectively. The finger showed good perfusion, with a capillary refill time of less than two seconds. The subjective sensation in the finger was rated as 10/10, with 0 representing numbness and 10 representing normal sensation. Magnetic resonance imaging (MRI) revealed features of tenosynovitis involving the flexor tendon (Figure 1 and Figure 2).

**Figure 3:**
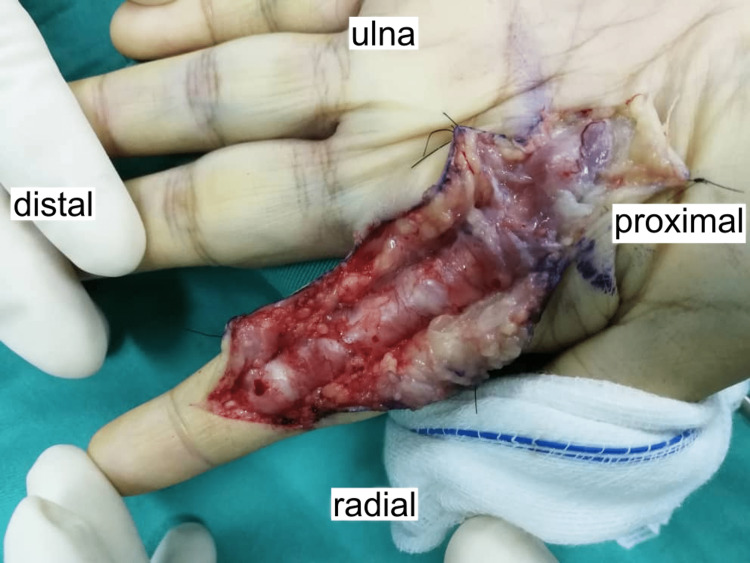
Intraoperative picture prior to the debridement. Intraoperative findings of abundant granulomatous tissue adhered to the flexor tendon sheath.

Hyper-intensified signals were observed around the flexor tendon, extending from the proximal phalanx to the midshaft of the second metacarpal bone. As medical therapy had failed, a decision was made to proceed with surgical intervention. During the exploration of the left index finger, it was observed that the synovial sheath of the flexor tendon was thick and unhealthy from the mid-palm to the A4 pulley (Figure 3).

**Figure 4:**
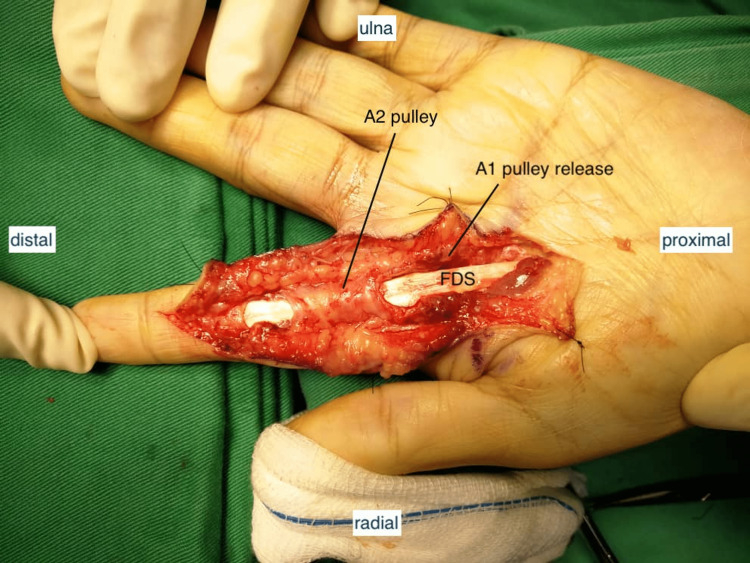
Intraoperative picture post debridement. A2 and A4 pulley were preserved. Other annular and cruciate pulley systems which adhered to the granulomatous tissue along the flexor tendon of the left index finger were excised. FDS: flexor digitorum superficialis

**Figure 5:**
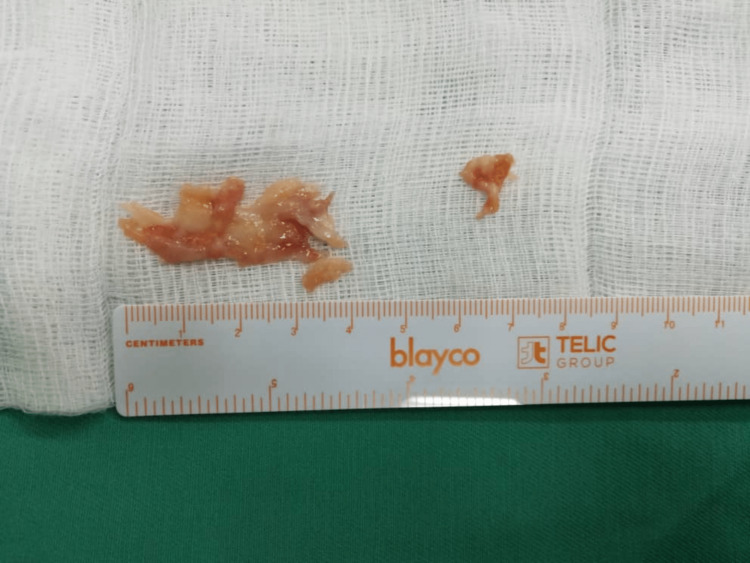
Figure showing the excised granulomatous tissue. Abundant granulomatous tissue adhered to the flexor tendon sheath.

Adhesions were found between the flexor digitorum superficialis (FDS) and the flexor digitorum profundus (FDP). The passive range of motion (ROM) of the index finger was limited due to resistance at the A1, A2, and A4 pulleys. No signs of an active infection were present. The synovial sheath was excised, and the A1 pulley was released (Figure 4 and Figure 5).

**Figure 6:**
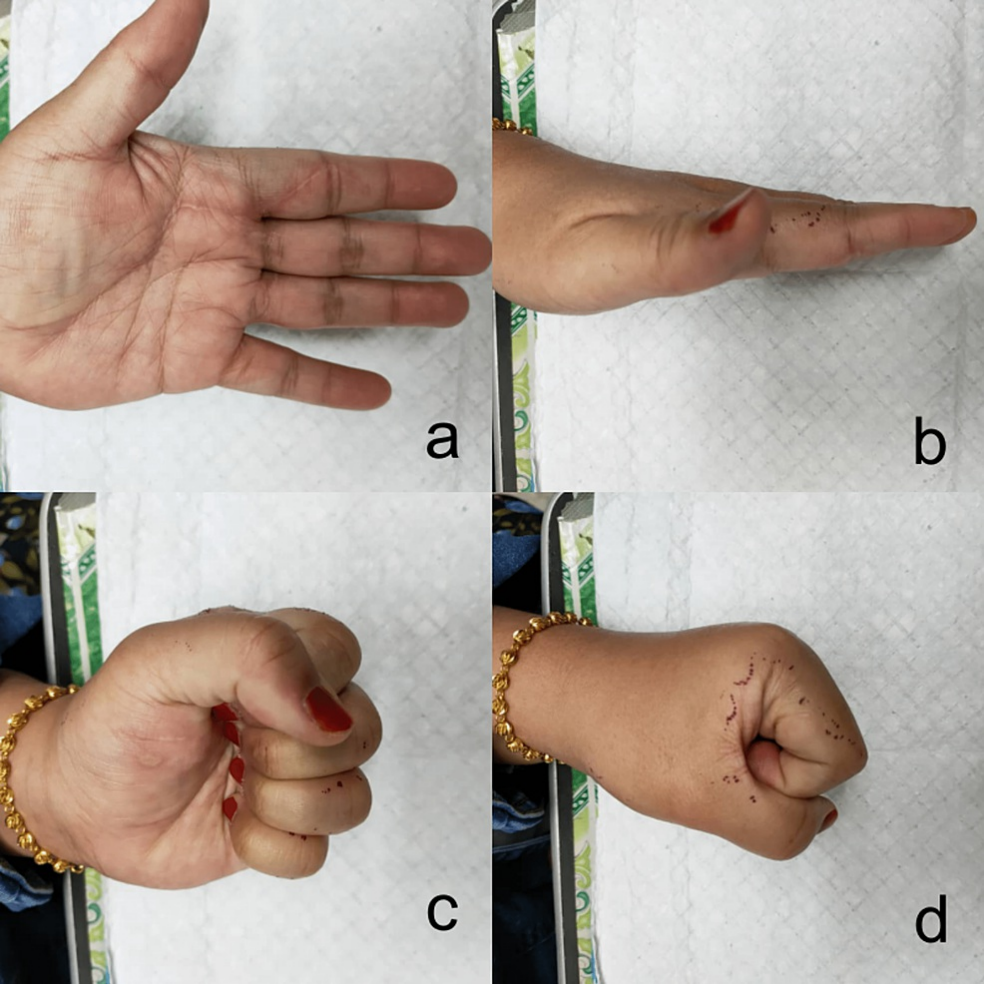
Figure showing the range of motion of left hand one month after the operation. Figure 6A and 6B demonstrate a well-healed surgical scar, resolved left index finger swelling, and full extension of the left index finger. Figure 6C and 6D show full flexion of the left index finger and a complete power grip.

Histopathology studies confirmed granulomatous synovitis. Tests for tuberculosis came back negative, and biochemical investigations did not suggest the presence of connective tissue disease. Following the debridement, the patient experienced significant improvement in pain and regained normal range of motion in her left index finger within one month (Figure 6).

## Discussion

To the best of our knowledge, this is the first reported case of chronic flexor tenosynovitis caused by CSD. Although uncommon, one should remain vigilant regarding the possibility of CSD contributing to chronic inflammation in the musculoskeletal system, particularly when assessing patients with a history of contact with cats.

In comparison to our reported case, the onset of typical pyogenic flexor tenosynovitis is acute. Clinically, an acute flexor tenosynovitis of the finger presents with Kanavel's signs [5]. Initiation of broad-spectrum antibiotics and prompt surgical debridement are often necessary to control the infection. Delayed treatment can lead to detrimental complications, including gangrene of the finger. Staphylococcus aureus is the most commonly isolated organism from tissue cultures in cases of acute flexor tenosynovitis [5]. From our research through PubMed, cases of chronic flexor tenosynovitis caused by tuberculous and non-tuberculous mycobacteria have been reported [6]. In most cases of non-tuberculous mycobacterial infection, patients usually recall a history of penetrating injury. Extensive tenosynovectomy and prolonged antibiotic treatment for up to five months are required in the reported cases.

Cats serve as the primary reservoir for Bartonella henselae [7]. It's important to note that not all cats infected with Bartonella henselae show signs of illness, making it challenging to identify infected animals. The chief feature of CSD is self-limited regional lymphadenopathy. However, involvement of visceral organs, neurologic systems, and ocular systems has been reported. Initiation of antibiotics is recommended in CSD in the presence of lymphadenitis. Studies have shown antibiotics shorten the duration of symptoms and reduce the risk of serious complications [3]. Azithromycin is the first-line therapy for CSD with mild lymphadenopathy [2]. Combination regimens can be prescribed for more severe cases.

Diagnosing CSD-related chronic flexor tenosynovitis is challenging due to the indolent course of the disease. Patients typically complain of dull pain and diffuse swelling, as seen in our reported case. The symptoms are more tolerable compared to tenosynovitis from other causes. Unfortunately, Kanavel's signs are not reliable for diagnosing CSD-related chronic flexor tenosynovitis. As a result, treatment initiation is often delayed.

Although CSD-related chronic flexor tenosynovitis does not lead to systemic sepsis or gangrene of the finger, it can cause significant morbidity in patients. Atypical and musculoskeletal manifestations of CSD have been described [8]. Most reported cases of CSD are self-limiting, and antibiotics are often not required [9]. There is no report on the role of surgery in these studies. However, in our case, the patient did not respond to antibiotics, and surgical debridement of the diseased finger successfully eliminated her chronic pain and restored finger ROM. Open debridement was performed in our case, and the role of limited incision drainage remains unknown.

## Conclusions

The history of cat contact is highly valuable when treating patients with chronic flexor tenosynovitis of the finger. A high index of suspicion is paramount to reaching a diagnosis. Early recognition of the disease can reduce patient morbidity and potentially avoid the need for extensive debridement. Surgical debridement of the finger for chronic flexor tenosynovitis caused by CSD might be beneficial in cases resistant to antibiotics. More effort should be made to raise awareness regarding the infections carried by domestic pets, as neglecting these diseases can cause significant morbidity.
